# Synergistic Effects of Pressure, Temperature, CO_2_ Flow Rate and Co-Solvent on Bioactive Contents of Thai Fingerroot (*Boesenbergia rotunda* (L.) Mansf.) Extracts

**DOI:** 10.3390/foods14132189

**Published:** 2025-06-23

**Authors:** Fahmi Ilman Fahrudin, Suphat Phongthai, Tri Indrarini Wirjantoro, Pilairuk Intipunya

**Affiliations:** 1 PhD Program in Food Science and Technology (International Program), Division of Food Science and Technology, Faculty of Agro-Industry, Chiang Mai University, Chiang Mai 50100, Thailand; fahmi_fahrudin@cmu.ac.th; 2 Division of Food Science and Technology, Faculty of Agro-Industry, Chiang Mai University, Chiang Mai 50100, Thailand; suphat.phongthai@cmu.ac.th (S.P.); triindrarini.w@cmu.ac.th (T.I.W.); 3 Cluster of High Value Products from Thai Rice and Plants for Health, Faculty of Agro-Industry, Chiang Mai University, Chiang Mai 50100, Thailand

**Keywords:** fingerroot, supercritical fluid extraction, pinocembrin, pinostrobin, antioxidants

## Abstract

This study investigated the use of supercritical carbon dioxide (CO_2_) to extract bioactive compounds from Thai fingerroot (*Boesenbergia rotunda*), focusing on the effects of pressure, temperature, CO_2_ flow rate, and ethanol co-solvent concentration. A central composite design within a response surface methodology framework was employed to optimize the total extraction yield, total phenolic content (TPC), and total flavonoid content (TFC). Conventional ethanol maceration was used as a benchmark. High-performance liquid chromatography identified the major compounds in the extracts, such as pinostrobin and pinocembrin. The results showed that the yield, TPC, and TFC increased with higher pressure, CO_2_ flow rate, and co-solvent levels, whereas higher temperatures had a negative effect (*p* ≤ 0.05). Interactions between pressure and temperature favored the yield and TPC but not TFC. The optimal conditions—250 bar, 45 °C, 3 L/min CO_2_ flow rate, and 100% ethanol—produced a yield of 28.67%, TPC of 354.578 mg GAE/g, and TFC of 273.479 mg QE/g. These values exceeded those obtained using conventional extraction (9.91% yield, 332.86 mg GAE/g TPC, and 77.57 mg QE/g TFC at 60 min). The regression models showed strong predictive accuracy (R^2^ > 0.9). Pinostrobin and pinocembrin were the dominant phenolic compounds. These findings demonstrate the superior efficiency of supercritical CO_2_ extraction for isolating phenolic compounds from *B. rotunda*.

## 1. Introduction

*Boesenbergia rotunda*, also known as fingerroot or Krachai Khao (common name in Thai), is a member of the ginger family (*Zingiberaceae*). This rhizome has various medicinal and culinary uses, especially in Southeast Asia. It is also rich in bioactive compounds such as flavonoids and essential oils, which have been shown to have various health benefits [1]. The phytoconstituents found in the rhizomes of *B. rotunda* are broadly categorized into two principal groups: (1) flavanones, including compounds like alpinetin, pinostrobin, and pinocembrin, and (2) chalcones, such as boesenbergin, cardamonin, panduratin A, and 4-hydroxypanduratin A [2]. These compounds have been found to possess anti-allergic, antibacterial, anticancer, anti-inflammatory, antioxidant, and anti-ulcer properties [3]. Recently, a study by Kanjanasirirat et al. [4] has found that fingerroot extract and its phenolic compounds show the most significant results in potent anti-SARS-CoV-2 activity compared to other Thai medicinal plants. Phenolic compounds constitute a class of bioactive compounds that have been extensively examined by the scientific community. These compounds are recognized for their well-documented health advantages, emphasizing the significance of including them in the daily diet to support overall well-being. Rich in phenolics, these molecules offer health benefits by neutralizing harmful free radicals. Their antioxidant effects are attributed to the donation of a hydrogen atom, an electron, or a series of interactions, including metal ion binding [5].

To obtain these valuable compounds, several extraction methods have been explored. Solvent-based extraction has been the predominant approach for isolating bioactive compounds from fingerroot [6,7]. Among these, maceration, a simple form of solid-liquid extraction, remains widely used, particularly for extracting herbal materials. The factors influencing solvent-based extraction include the solvent type, extraction time, temperature, solvent-to-sample ratio, agitation, and separation technique. While ethanol, acetone, and water are commonly used in the food industry to extract polyphenols [8,9], methanol is often employed in research studies due to its high extraction efficiency despite its toxicity and unsuitability for food applications. The reported extraction yields were 8.8% with acetone, 10% with ethanol, 11% with methanol, and 14% with water [7].

Although maceration is simple, it often results in low extraction yields and limited bioactive compound recovery [10,11]. Furthermore, it requires extended extraction times and large volumes of solvents, making it inefficient for industrial use. To overcome these drawbacks, supercritical carbon dioxide (CO_2_) extraction (SFE) has emerged as a promising alternative in food processing applications. SFE offers several advantages, including lower extraction temperatures, shorter extraction times, the absence of toxic solvent residues, higher extraction efficiencies, and enhanced product quality. Moreover, it aligns with sustainability goals due to its reduced energy consumption and environmental impact [12,13,14]. Recent studies have highlighted the growing adoption of SFE in food industry applications, particularly for extracting high-value compounds such as essential oils, omega-3 fatty acids, and phytochemicals while meeting stringent food safety standards [15,16]. These advances further emphasize SFE’s role in sustainable food processing and the development of functional ingredients. SFE is particularly suitable for recovering diverse constituents, especially non-polar organic compounds. This efficacy stems from the mild extraction conditions facilitated by CO_2_’s low critical temperature and pressure. These characteristics render this process highly applicable to various applications [17]. Although there are examples of successful SFE extraction of abundant bioactive compounds, such as pinostrobin and pinocembrin, from *Populus nigra* L., which significantly enhances the total phenolic content and antioxidant activity [18], there is insufficient data on fingerroot extraction; hence, the optimization of SFE conditions awaits exploration.

Given this gap, exploring the SFE of *B. rotunda* is essential for unlocking its potential in the food, pharmaceutical, and cosmetic sectors. Rich in bioactive compounds with antioxidant, anti-inflammatory, and antimicrobial properties [19,20], *B. rotunda* aligns with the growing consumer demand for safe, natural, and effective ingredients. SFE not only enhances the yield and purity of these compounds but also addresses the challenges associated with solvent-based methods [21]. The multifunctional bioactivity of *B. rotunda* makes it particularly valuable for the development of functional foods, nutraceuticals, and cosmetic products.

Therefore, this study aimed to investigate the synergistic effects of pressure, temperature, CO_2_ flow rate, and co-solvent concentration on optimizing the supercritical fluid extraction (SFE) process for extracting potential bioactive compounds from *B. rotunda*. These extraction factors were systematically varied and optimized to enhance the extraction efficiency. Additionally, the extraction yields and key chemical properties of the SFE extracts were compared with those obtained from conventional solvent-based extraction to evaluate the feasibility and potential advantages of the SFE process for product development and utilization of the extracted products.

## 2. Materials and Methods

### 2.1. Materials and Reagents

*B. rotunda* was bought from the Chiang Mai University Agri Shop and kept in a refrigerator at 4 °C for 24 h at the Division of Food Science and Technology, Faculty of Agro-Industry, Chiang Mai University, Thailand. The rhizome was cleaned and sliced before freeze-drying at an initial temperature of −40 °C under 20 Pa pressure for 24 h. After drying, the rhizome was ground into a fine powder and sieved through a mesh filter (0.3 mm). Pinostrobin and pinocembrin standards were purchased from Shanghai Macklin Biochemical Co., Ltd. (Shanghai, China), Acetonitrile HPLC Grade (RCI Labscan Ltd., Bangkok, Thailand), and ortho-phosphoric acid (Merck KGaA, Darmstadt, Germany).

### 2.2. Maceration Extraction

The conventional extraction method for fingerroot utilizes solvent extraction, as described by Atun et al. [10], with some modifications. Ethanol was chosen as the extraction solvent because it showed significant results in a previous study [7,22]. Ethanol (95% concentration) was used as the extraction solvent at a solvent-to-solid ratio of 1:1, as demonstrated by Lee et al. [23], who showed its efficacy in obtaining the most bioactive compounds from the fingerroot. The freeze-dried sample (20 g) was macerated with 100 mL of ethanol at a controlled temperature of 27 °C for 40, 60 and 80 min, according to Fahrudin et al. [24]. The mixture was then filtered using Whatman^®^ filter paper No. 4 and evaporated to dryness using a rotary evaporator. The resulting extracts were stored at 4 °C until further analysis and processing, as per the method outlined by [25].

### 2.3. Supercritical CO_2_ Fluid Extraction

All experiments were performed using the Speed™ SFE-2 system (Applied Separations Inc., Allentown, PA, USA) according to the manufacturer’s instructions. Carbon dioxide (99% purity) was obtained from Iextract, Northern Science Park, Chiang Mai University, Thailand. The operational procedure involved placing approximately 20 g of fingerroot powder into a 24-mL extractor fitted with a filter film and wool on both ends to prevent particle flushing. After loading the extractor into the vessel, the system was charged with CO_2_. The apparatus was cooled for one hour to optimize the extraction before reaching the designated pressure and temperature settings. Various experimental conditions were examined, including temperatures ranging from 35 to 55 °C, pressures from 200 to 300 bar, CO_2_ flow rates from 1 to 3 L/min, and ethanol as a co-solvent at varying solid-to-liquid ratios. To facilitate conversion of the volumetric CO₂ flow rates (L/min) to mass flow rates (kg/min), density correction factors based on CO₂ density at the corresponding pressures and temperatures can be applied. The CO₂ density values can be obtained from the NIST Chemistry WebBook (https://webbook.nist.gov/chemistry/fluid/) or calculated using standard equations of state for CO₂. This allows reproducibility and comparison across different experimental setups.The co-solvent ratios experimented in this study were 1:0 (0%), 2:1 (50%), and 1:1 (100%), representing the weight ratio of the dried sample to ethanol. In this study, ethanol was employed by pre-mixing it with the dried sample before loading it into the extraction vessel rather than being continuously introduced during the extraction. The mixture was held under static conditions while the system was pressurized and heated to the desired temperature prior to extraction. This ethanol soaking step was intended to enhance the solubilization of phenolic compounds before the supercritical CO_2_ extraction. Subsequently, dynamic extraction was performed by introducing CO_2_ at a controlled flow rate for 30 min. The 30 min extraction time was selected based on preliminary experiments and supported by previous studies, which demonstrated that this duration is generally sufficient for achieving efficient extraction of bioactive compounds while balancing the process efficiency and operational practicality. The CO_2_-infused extract was collected from the outlet. The fingerroot extract was weighed, transferred to a dark bottle, and stored in a refrigerator (4 ^o^C) until further analysis.

### 2.4. Experimental Design

A response surface methodology with a central composite design (CCD) was applied to optimize the extraction of fingerroot. This optimization was performed after the initial determination of the extraction variable range through single-factor tests. The study investigated four independent variables (X_1_ (temperature), X_2_ (pressure), X_3_ (CO_2_ flow rate), and X_4_ (percentage of co-solvent to sample mass) at three distinct levels. For the statistical calculations, the variables were coded according to Equation (1).

(1) $$x_{i}=(X_{i}-X_{0})/∆X_{i}$$

where *x_i_* is a coded value of the variable; $X_{i}$ is the actual value of a variable; $X_{0}$ is the actual value of the $X_{i}$ on the center point; $∆X_{i}$ is the step change value. Table 1 lists the ranges of the independent variables and their levels. The experimental design consisted of 30 experiments, including six center points according to the CCD, as shown in Table 2.

Design Expert version 13 (Stat-Ease Inc., Minneapolis, MN, USA) software was used to investigate the results, build and evaluate models, and plot three-dimensional response surface curves based on Equation (2).

(2) $$Y={\;\beta }_{0}+\beta_{1}A+\beta_{1}B+{\;\beta }_{1}C+\beta_{1}D+{\;\beta }_{11}A^{2}+\beta_{22}B^{2}+\beta_{33}C^{2}+\beta_{44}D^{2}+\beta_{12}AB+\beta_{13}AC+\beta_{14}AD+\beta_{23}BC+\beta_{24}BD+\beta_{34}CD$$

The equation encompasses diverse terms, including the intercept term (*β*_0_), linear coefficients (*β*_1_, *β*_2_, *β*_3,_ and *β*_4_), quadratic coefficients (*β*_11_, *β*_22_, *β*_33,_ and *β*_44_), and interaction coefficients (*β*_12_, *β*_13_, *β*_23,_ and *β*_24_). These coefficients are associated with the measured response (Y) and coded independent variables (*A* (pressure), *B* (temperature), *C* (CO_2_ flow rate), and *D* (percentage of co-solvent)). Three metrics were examined to assess the model fit: R-squared (coefficient of determination), predicted R-squared, and adjusted R-squared. Fisher’s F-test was used to determine the statistical significance of the model. To visualize the connection between the independent and dependent variables, 3D graphs and corresponding contour plots were generated.

### 2.5. Total Yield Analysis

The extracts obtained from the SFE process were evaporated to dryness and weighed, and the total yields were calculated using Equation (3) [26]. The temperature of the rotary evaporator was set at 50 °C under a vacuum of 200 mbar. The extracts were concentrated as the solvent evaporated.

(3) $$Total\;Yield\;\left(\%\right)\;=\;\left[\frac{Weigth\;of\;Extract}{Weight\;of\;Sample}\right]\times 100$$

### 2.6. Total Phenolic Content (TPC) Analysis

The total phenolic content (TPC) was determined using the Folin-Ciocalteu reagent, as described by Nawaz et al. [27]. A 500 μL sample solution was mixed with 2.5 mL of 10% *v/v* Folin-Ciocalteu reagent. After 5 min of incubation at room temperature, 2 mL of 7.5% *(v/v)* sodium carbonate was added. The reaction mixture was left for 45 min at 28 °C, and the absorbance was measured at 760 nm using a GENESYS™ 180 UV-Vis spectrophotometer (Thermo Fisher Scientific, Waltham, MA, USA). A gallic acid standard curve was used to calculate the TPC, prepared by diluting a 1% *m*/*v* gallic acid solution to concentrations of 0.1, 0.5, 1.0, 2.5, and 5.0 mg/mL. The results were expressed as milligrams of gallic acid equivalents per gram (mg GAE/g), calculated from the gallic acid standard calibration curve using Equation (4).

(4) $$TPC\;\left(mg\;GAE/g\right)\;=(\frac{Gallic\;acid\;\left(mg/mL\right)\times Volume\;of\;Extract\;\left(mL\right)}{Mass\;of\;Extract\;\left(g\right)})$$

### 2.7. Total Flavonoid Content (TFC) Analysis

The TFC of the extracts was determined using an aluminum chloride complex-forming assay, following the method described by Velioglu et al. [28]. Quercetin was the standard, and TFC was expressed in milligrams of quercetin equivalents per gram (mg QE/g). A quercetin standard solution was prepared in ethanol at concentrations of 0.1, 0.5, 1.0, 2.5 mg/mL, and 5.0 mg/mL. For each dilution, 100 µL of quercetin solution was mixed with 500 µL of distilled water and 100 µL of 5% (m/v) sodium nitrate solution, and the mixture was incubated for 6 min. Next, 150 µL of 10% (m/v) aluminum chloride solution was added and incubated for 5 min, followed by 200 µL of 1 M sodium hydroxide. The absorbance of the reaction mixture was measured at 510 nm using a GENESYS™ 180 UV-Vis spectrophotometer (Thermo Fisher Scientific, USA). TFC was calculated using a quercetin standard calibration curve, following Equation (5).

(5) $$TFC\;\left(mg\;QE/g\right)=\;(\frac{Quercetin\;\left(mg/mL\right)\times Volume\;of\;Extract\;\left(mL\right)}{Mass\;of\;Extract\;\left(g\right)})$$

### 2.8. Extract Composition Analysis

High-performance liquid chromatography (HPLC) separations were performed on a 250 mm × 4.6 mm, 5 µm Agilent Zorbax SB-C18 column (Agilent 1260, Agilent Technologies, Santa Clara, CA, USA). Flavonoid analysis was performed using gradient elution with a mobile phase consisting of 0.1% phosphoric acid (solvent A) and acetonitrile (solvent B). The elution program was as follows: 20% A/80% B from 0 to 35 min, then gradually returning to 80% A/20% B between 35 and 45 min, and maintaining at 80% A/20% B until 50 min. The total run time was 50 min, with a flow rate of 1 mL/min, and detection was performed at a wavelength of 256 nm. The 10 μL samples were injected, and flavonoids were identified by comparing the retention time of the absorption spectra with those of the reference standards. The quantification of pinocembrin and pinostrobin in the extracts was performed using a standard calibration method applicable within the range of 0.5 to 50 ppm. The data were acquired and expressed as µg of each compound per gram of dry extract.

## 3. Results and Discussion

### 3.1. Extraction Yield, Phenolic and Flavonoid Contents from Maceration Extraction

The extraction yield of fingerroot, total phenolic content, and total flavonoid content are presented in Table 3. The highest yield of 9.91% was obtained at an extraction time of 60 min (*p* ≤ 0.05), whereas extraction using shorter (40 min) or longer (80 min) extraction times showed no significant difference (*p* > 0.05). A maceration time of 60 min may be sufficient to allow mass diffusion and effective transport of soluble compounds from the fingerroot powder into the ethanol solvent during solid-liquid extraction, facilitated by the small particle size of the powder. Extraction time did not significantly affect the total phenolic content. However, a higher TPC was obtained when the maceration time was 60 min (*p* > 0.05), showing a value of 332.86 mg GAE/g. Evidence suggests that prolonged extraction times do not significantly enhance yields compared to optimized shorter durations, as reported in previous studies, where variations in extraction time had minimal impact on phenolic content during maceration extraction [29]. TFC was the highest (109.39 mg QE/g) when extracted for 40 min (*p* ≤ 0.05), whereas increasing the extraction time to 60 and 80 min did not significantly change the TFC (*p* > 0.05). From the TPC and TFC results, 60 min extraction time yielded the highest TPC, whereas 40 min yielded the highest TFC. This difference may be attributed to the greater oxidative stability of TPC during maceration, while TFC is more prone to degradation with prolonged oxygen exposure. For example, Alencar et al. [30] reported that the TPC increases during the early stages of maceration and remains stable over time. In contrast, Sithisarn et al. [31] observed that TFC is more susceptible to oxidation, leading to reduced antioxidant activity during prolonged extraction. These findings suggest that while maceration efficiently extracts phenolics, TPC remains stable, whereas TFC may decline under prolonged maceration conditions [32]. Therefore, it is recommended that 60 min of maceration is adequate for recovering the extractable bioactive compounds from the fingerroot.

Previous studies have revealed that oxidative stability is strongly associated with total phenols, carotenoids and chlorophylls [33]. Furthermore, an increase in total phenolic content is consistently linked to heightened antioxidant capacity, suggesting that phenolic compounds can mitigate oxidative damage more effectively than flavonoids [34]. Therefore, TPC is positioned as a more stable compound under oxidative stress. In contrast, total flavonoid content seems more variable under thermal processes. For instance, blanching and high-temperature treatments often lead to a decrease in flavonoid concentrations [35]. While flavonoids provide antioxidant benefits, their susceptibility to degradation has been noted in multiple studies. For example, thermal processing has been shown to significantly decrease TFC in various fruits and vegetables [36].

### 3.2. Extraction Yield and Bioactive Contents of Fingerroot Extracts from SFE

The synergistic effects of pressure, temperature, CO_2_ flow rate, and co-solvent addition on fingerroot extraction varied among the 30 extraction trials. The experimental responses, including extraction yield, total phenolic content (TPC), and total flavonoid content (TFC), as summarized in Table 4, were analyzed using Analysis of Variance (ANOVA), resulting in the non-linear regression parameters presented in Equations (6)–(8). Among the parameters studied, temperature and pressure were particularly significant as they directly influenced the density of the solvent. Increasing pressure, CO_2_ flow rate, and co-solvent positively influenced the extraction yield, TPC, and TFC, while increasing temperature slightly reduced these responses. However, the combined effects of pressure and temperature favored the yield and TPC. In addition to pressure−temperature, there were slight adverse effects of combined factors on TPC. Although the individual temperature factor showed a slight downturn in TFC, its combined effect with the CO_2_ flow rate and co-solvent revealed favorable results.

(6) Yield = 21.73 + 1.02*A* − 0.38*B* + 0.13*C* + 11.56*D* − 1.75*A*^2^ + 0.40*B*^2^ + 1.63*C*^2^ − 2.25*D*^2^ + 0.13*AB* − 0.12*AC* − 0.77*AD* − 0.075*BC* − 0.24*BD* − 0.28*CD*

(7) TPC = 443.87 + 4.36*A* − 25.30*B* + 2.31*C* + 34.81*D* − 110.21*A*^2^ − 31.31*B*^2^ + 12.94*C*^2^ − 31.01*D*^2^ + 13.49*AB* − 11.21*AC* − 11.02*AD* − 4.76*BC* − 12.94*BD* + 0.7250*CD*

(8) TFC = 305.80 + 27.34*A* − 2.91*B* + 6.76*C* + 75.19*D* − 103.33*A*^2^ + 48.92*B*^2^ − 5.83*C*^2^ − 57.33*D*^2^ − 9.06*AB* − 5.36*AC* − 6.25*AD* + 6.01*BC* + 3.13*BD* + 4.28*CD*

The effects of the treatment factors on the measured responses were closely linked to the properties of the supercritical CO_2_ and the chemical nature of the extracted compounds. Altering the temperature and pressure modifies the solvent density, particularly within the critical region, where slight variations in these parameters produce substantial changes in the CO_2_ density and solvent power. This phenomenon explains the observed increase in the extraction yield with increasing pressure up to a certain point. However, at pressures above 250 bar, the extraction yield plateaued or slightly decreased. This trend may be attributed to excessive pressure, causing compaction of the extraction bed, potentially hindering CO_2_ flow and reducing mass transfer efficiency [37]. The formation of preferential channels within the extraction matrix could also contribute to uneven extraction, leaving parts of the sample under-extracted. Thus, the experimental results aligned with the theoretical expectation that while higher pressures can initially enhance extraction, excessively high pressures can introduce mass transfer limitations that counteract the benefits of the increased solvent density.

Table 5 summarizes the statistical tests used to evaluate the extraction of the fingerroot models, including the F-test and its corresponding probabilities. The analysis of variance (ANOVA) for the quadratic regression models revealed high values of correlation coefficients (R^2^) for models (6)–(8) above 0.90, which means that the models were significant or strongly correlated in representing the data. The R^2^ values of 0.9817 for the extraction yield model and 0.9330 for the TPC model indicate that the models provide a good fit to the experimental data, particularly for extraction yield. While the extraction yield model demonstrated excellent predictive capability, the TPC model showed a reasonably strong correlation, although with slightly greater variation between the predicted and observed values. These results suggest that the developed models are reliable for describing and predicting extraction behavior under the tested conditions. The differences of 0.02 and 0.07 indicate that the models were highly significant for the extraction yield and TPC, respectively. These results suggest the model’s ability to make accurate predictions within a tested range. A “lack of fit” F-value of 3.61 and 3.23 for both responses confirmed the model’s suitability for analyzing the factors investigated in this study. Although the differences in R^2^ compared to 1 were 0.1 for TFC, the *p*-values for these models were less than 0.05, indicating that the models were strongly significant.

The ANOVA results (*p* < 0.0001) confirmed the reliability of the model. Interactions between variables, such as pressure−temperature or flow rate-co-solvent, also play significant roles. The equation indicates that higher flow rates and co-solvent ratios improve flavonoid content, but only up to an optimal threshold (e.g., ~2 L/min, as shown in supercritical extraction studies). Beyond this point, further increases yield diminishing returns due to saturation in mass transfer efficiency [38]. The principle of enhanced mass transfer through a higher flow rate remains applicable when targeting flavonoids, which are typically more polar and may require more effective solvent renewal during extraction [39]. They similarly found that balancing multiple factors is key to efficient extraction. At the same time, adding a co-solvent (or entrainer), typically ethanol, is crucial due to the inherently low polarity of CO_2_. Ethanol increases the overall polarity of the extraction medium, thereby improving the solubility of polar flavonoids and other bioactive compounds [40]. For flavonoids, which have limited solubility in pure supercritical CO_2_ (SC-CO_2_), the addition of a co-solvent, such as ethanol, facilitates their extraction by forming hydrogen bonds with flavonoid molecules, thereby enhancing their dissolution [41]. This finding is consistent with previous studies reporting that the optimized use of ethanol as a co-solvent improves the extraction yield of bioactive compounds from plant materials [42]. Furthermore, based on the regression model and ANOVA results, pressure was identified as the most significant factor influencing extraction efficiency.

**Figure 1:**
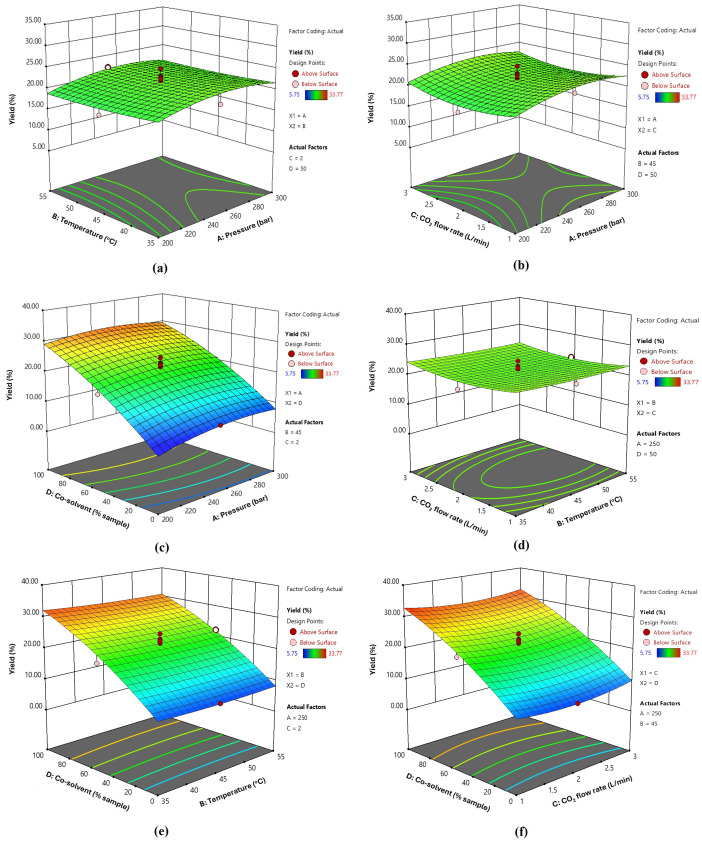
Response surface 3D plots of independent variables on extraction yields of fingerroot extracts, interactions between factors: (**a**) temperature and pressure (AB); (**b**) CO_2_ flow rate and pressure (AC); (**c**) co-solvent and pressure (AD); (**d**) temperature and CO_2_ flow rate (BC); (**e**) temperature and co-solvent (BD); (**f**) CO_2_ flow rate and co-solvent (CD).

**Figure 2:**
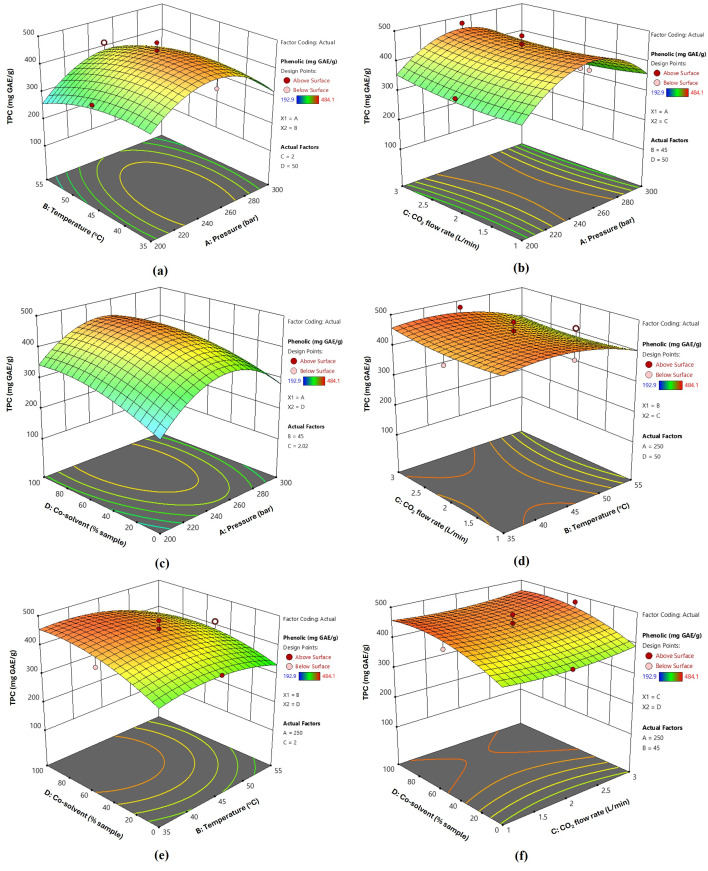
Response surface 3D plots of independent variables on the TPC of fingerroot extracts, interactions between factors: (**a**) temperature and pressure (AB); (**b**) CO_2_ flow rate and pressure (AC); (**c**) co-solvent and pressure (AD); (**d**) temperature and CO_2_ flow rate (BC); (**e**) temperature and co-solvent (BD); (**f**) CO_2_ flow rate and co-solvent (CD).

**Figure 3:**
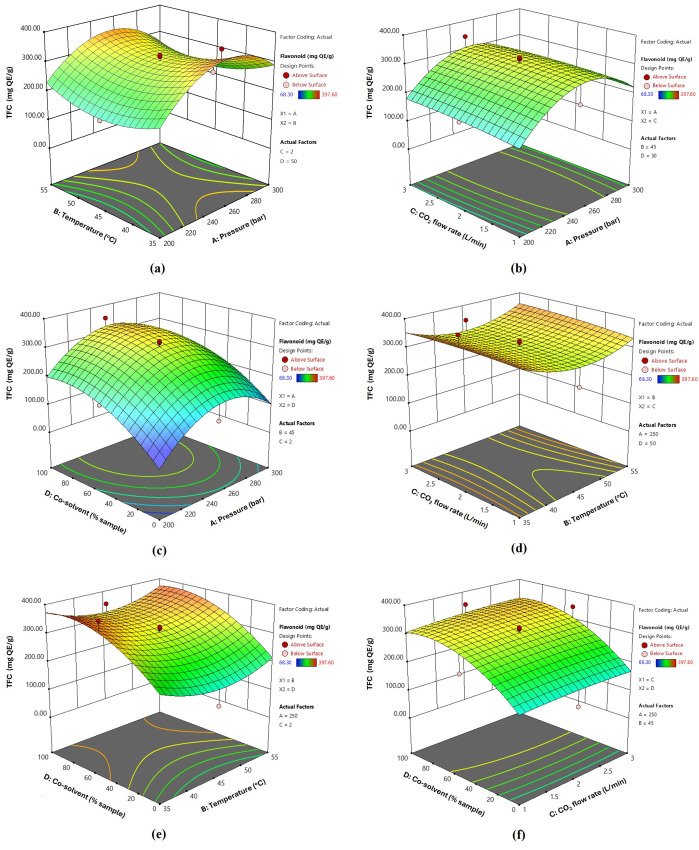
Response surface 3D plots of independent variables on TFC of fingerroot extracts, Interactions between factors: (**a**) temperature and pressure (AB); (**b**) CO_2_ flow rate and pressure (AC); (**c**) Co-solvent and pressure (AD); (**d**) temperature and CO_2_ flow rate (BC); (**e**) temperature and co-solvent (BD); (**f**) CO_2_ flow rate and co-solvent (CD).

All CO_2_-SFE factors contributed to variations in the extraction performance and quality attributes of the extract. Using Design Expert 13, contour and 3D surface plots were generated to predict the optimal extraction of bioactive compounds from fingerroot using the CO_2_-SFE process. Figure 1, Figure 2 and Figure 3 illustrate the variations in the yield, TPC, and TFC of the extracts under different extraction pressures, temperatures, CO_2_ flow rates, and co-solvent conditions. The three-dimensional response surface plots illustrate the relationships between two continuous variables and the extraction of bioactive compounds from fingerroot, with other variables maintained at their central values (0 levels of the testing range). Analysis of the 3D surface plots revealed that the addition of ethanol, used as a soaking agent prior to extraction, significantly influenced the extraction yield. In this study, ethanol was not introduced as a continuous co-solvent during the supercritical fluid extraction process but was pre-mixed with the dried sample before pressurization. Consequently, the ethanol concentration was not actively controlled during extraction, and the phase equilibria likely evolved throughout the process as ethanol partitioned into the CO_2_ phase. Despite this, ethanol soaking effectively enhanced the solubilization of phenolic compounds, contributing to improved extraction yield.

In contrast, the phenolic content results showed that the extraction factors, such as pressure and temperature, had a significant impact on the extraction yield. This demonstrates that pressure and temperature significantly influenced the TPC of fingerroot (Figure 2a,b), exhibiting a noticeable curve on the 3D plot surface. The increase in carbon dioxide flow rate and co-solvent addition also influenced the total phenolic content, as depicted in Figure 2c,d. Simultaneously, Figure 2e,f indicates that additional co-solvent contributes significantly to an increase in the phenolic content of fingerroot. These conditions were consistent with those of previous studies, which mentioned that the combination of solvents accelerated the extraction process, reduced the usage of CO_2_, and enhanced the extraction yield.

Figure 3 illustrates the relationship between the TFC and extraction parameters. In the present study, the flavonoid content tended to increase with pressure up to a certain point, after which further increases led to a decline in extraction efficiency, suggesting the existence of an optimal pressure range. Although similar trends have been reported for other plant extractions [43,44], differences in matrix composition and compound polarity mean that such comparisons should be interpreted with caution. These findings underscore the importance of optimizing the extraction conditions, specifically for *B. rotunda*, to maximize flavonoid recovery. At 50 °C, pressures above 200 bar were also observed to reduce the extraction efficiency of phenolic compounds, which may be attributed to changes in solvent density or reduced solubility and selectivity under these conditions. The curved shapes in the plots suggest that there is an optimal “middle point” for variables such as pressure and temperature to obtain the highest flavonoid yield. Similarly, studies on supercritical CO_2_ extraction have demonstrated that moderate temperatures can increase the vapor pressure and diffusivity of solutes, thus enhancing the solubility of flavonoid compounds [45]. In these cases, pressure levels in the range of approximately 200 to 350 bar combined with temperatures around 40–60 °C appear to be optimal, as these conditions maintain high solvent density and enhance mass transfer while minimizing the risk of thermal degradation [46].

The largest coefficient and significant quadratic effect were optimal between 250 and 350 bar. Temperature was the second significant factor, showing a moderate effect, with an optimal range of 40–60 °C. The flow rate and co-solvent ratio were less impactful but still significant. Key interactions like pressure-temperature (negative) and CO_2_ flow rate-co-solvent (positive) exist but are less dominant. For efficient fingerroot extraction, the extraction should prioritize pressure and temperature control, supported by a moderate flow rate and the use of a co-solvent.

### 3.3. Optimization and Validation of SFE Conditions for Fingerroot Extract

Based on the 3D response surface methodology shown in Figure 1, Figure 2 and Figure 3, the optimal extraction conditions were determined to be as follows: extraction pressure at 250 bar, temperature at 45 °C, carbon dioxide flow rate set at 3 L/min, and co-solvent concentration of 100% *w*/*v*. Under these specified conditions, the actual yield, TPC, and TFC were 28.67%, 354.578 mg GAE/g and 273.479 mg QE/g, respectively (Table 6). The additional ethanol concentration as a co-solvent substantially impacted the extraction yield. The investigation encompassed four parameters, and from the regression coefficients of the quadratic polynomial model in Table 4, as well as the information gleaned from the 3D response surface plots, it was determined that extraction pressure and the addition of a co-solvent were the most influential factors affecting the extraction of fingerroot oil.

To evaluate the predictive performance of the model, optimization was conducted at a pressure of 250 bar, temperature of 45 °C, CO_2_ flow rate of 3 L/min, and 100% (*w*/*w*) co-solvent ratio. Under these conditions, the model predicted an extraction yield of 32.51%, TPC of 463.60 mg GAE/g, and TFC of 328.91 mg QE/g. However, the corresponding experimental values were 28.67%, 354.58 mg GAE/g, and 273.48 mg QE/g, respectively. These results demonstrated deviations of 11.81% for yield, 23.52% for TPC, and 16.85% for TFC from the predicted values. Although some discrepancies were observed, especially for TPC, the experimental results confirmed the general predictive capability of the model, particularly regarding the extraction efficiency trends. The lower actual yields may be attributed to practical factors, such as mass transfer limitations and variations in the sample matrix during scaling. Overall, the findings affirm the model’s utility as a predictive and optimization tool while underscoring the importance of empirical validation to support its practical application in real extraction processes.

### 3.4. Comparison of Conventional Extraction and Supercritical Fluid Extraction (SFE)

A comparison between supercritical CO_2_ fluid extraction (SFE) and maceration extraction revealed that the extraction yield, TPC, and TFC obtained by SFE under optimum conditions were superior (28.67%, 354.578 mg GAE/g, 273.479 mg QE/g from SFE, respectively, as compared to 9.91%, 332.86 mg GAE/g, 77.57 mg QE/g from maceration, respectively). Furthermore, the SFE method demonstrated a shorter extraction time than that of the maceration process. This observation underscores the efficiency of SFE and highlights its potential to provide more significant results than conventional extraction methods, overcoming some of the limitations associated with traditional approaches.

### 3.5. Main Compounds in Boesenbergia Rotunda Extract

HPLC analysis revealed that pinocembrin and pinostrobin were the prominent compounds in *the B. rotunda* extract. These compounds are integral to the medicinal attributes of the plant. The pinocembrin and pinostrobin concentrations are shown in Table 7. The extraction of pinostrobin was favorable when the pressure, CO_2_ flow rate, and co-solvent were increased. At the same time, pinocembrin positively responded to increasing co-solvent and combined effects of pressure, co-solvent, and temperature-CO_2_ flow rate. The maximum concentrations of 62.10 mg/g and 115.28 mg/g were extracted at 250 bar, 45 ^o^C, 2 L/min CO_2_ flow rate, and 100% *v*/*w* co-solvent. However, under the optimized conditions based on yield, TPC, and TFC, the extract contained 32.628 mg/g pinocembrin and 82.655 mg/g pinostrobin. Supercritical fluid extraction was considered a beneficial method for effectively isolating pinocembrin and pinostrobin from fingerroot. This underscores the importance of extraction process factors in retrieving these valuable compounds.

Due to the disparate polarities of pinostrobin and pinocembrin, their extraction requires the use of distinct solvents. This consideration was addressed in this study by employing both polar and non-polar solvents. Ethanol, owing to its polar nature, facilitates the extraction of pinocembrin by attracting polar molecules. Conversely, carbon dioxide is adept at extracting pinostrobin because of its compatibility with the properties of the compound. Moreover, manipulating the pressure and temperature within the supercritical fluid extraction process is critical for achieving the requisite phase transitions and densities conducive to optimal extraction outcomes. This is consistent with the findings of a similar study by [47], in which temperature and pressure were the main parameters that influenced the increase in yield extraction.

## 4. Conclusions

Response Surface Methodology was effectively applied to optimize the supercritical CO_2_ extraction of *B. rotunda*. The substantial regression coefficients of the second-order polynomial suggest a robust fit of the model to experimental data. Analysis of Variance (ANOVA) results highlighted extraction pressure as the most influential factor impacting the phenolic compounds in supercritical CO_2_ extraction. The optimal conditions for SFE were identified as an extraction pressure of 250 bar, temperature of 45 °C, carbon dioxide flow rate of 3 L/min, and co-solvent concentration of 100%. Under these conditions, the extraction yield was the highest, and the TPC and TFC were optimal. Pinostrobin and pinocembrin were the main bioactive compounds in the extracts.

Supercritical carbon dioxide-extracted fingerroot presents a desirable profile abundant in bioactive compounds, rendering it suitable for utilization as a food additive and in pharmaceutical products across diverse industries. This research holds substantial significance as it sheds light on the broad spectrum of potential applications within the food and pharmaceutical sectors, expanding the understanding of its versatility and utility beyond traditional uses.

## Figures and Tables

**Table 1:** Extraction conditions from the response surface methodology with a central composite design.

Independent Variables	Symbol	Levels		
		−1	0	1
Pressure (Bar)	A	200	250	300
Temperature (°C)	B	35	45	55
Flow rate (L/min)	C	1	2	3
Co-solvent (% of sample mass)	D	0	50	100

**Table 2:** Independent variables and their levels used in the response surface design.

Run	Pressure (Bar)	Temperature (°C)	CO_2_ Flow Rate (L/min)	Co-Solvent (% of Sample Mass)
1	(1) 300	(1) 55	(−1) 1	(−1) 0
2	(−1) 200	(1) 55	(1) 3	(1) 100
3	(1) 300	(0) 45	(0) 2	(0) 50
4	(1) 300	(−1) 35	(−1) 1	(−1) 0
5	(0) 250	(0) 45	(0) 2	(0) 50
6	(0) 250	(0) 45	(0) 2	(0) 50
7	(0) 250	(0) 45	(1) 3	(0) 50
8	(1) 300	(1) 55	(1) 3	(−1) 0
9	(1) 300	(1) 55	(−1) 1	(1) 100
10	(−1) 200	(−1) 35	(−1) 1	(−1) 0
11	(−1) 200	(−1) 35	(−1) 1	(1) 100
12	(0) 250	(0) 45	(0) 2	(−1) 0
13	(1) 300	(1) 55	(1) 3	(1) 100
14	(0) 250	(0) 45	(0) 2	(0) 50
15	(0) 250	(0) 45	(0) 2	(0) 50
16	(0) 250	(0) 45	(0) 2	(1) 100
17	(0) 250	(0) 45	(−1) 1	(0) 50
18	(0) 250	(1) 55	(0) 2	(0) 50
19	(1) 300	(−1) 35	(1) 3	(1) 100
20	(−1) 200	(−1) 35	(1) 3	(−1) 0
21	(−1) 200	(0) 45	(0) 2	(0) 50
22	(−1) 200	(1) 55	(1) 3	(−1) 0
23	(−1) 200	(1) 55	(−1) 1	(−1) 0
24	(−1) 200	(−1) 35	(1) 3	(1) 100
25	(0) 250	(0) 45	(0) 2	(0) 50
26	(1) 300	(−1) 35	(1) 3	(−1) 0
27	(−1) 200	(1) 55	(−1) 1	(1) 100
28	(0) 250	(−1) 35	(0) 2	(0) 50
29	(0) 250	(0) 45	(0) 2	(0) 50
30	(1) 300	(−1) 35	(−1) 1	(1) 100

**Table 3:** Extraction yields and total phenolic and flavonoid contents of fingerroot maceration extracts.

Extraction Time (min)	Total Yield (%)	TPC (mg GAE/g)	TFC (mg QE/g)
40	9.00 ± 0.10 ^b^	276.16 ± 7.36 ^a^	109.39 ± 3.67 ^a^
60	9.91 ± 0.08 ^a^	332.86 ± 8.42 ^a^	77.57 ± 0.97 ^b^
80	9.12 ± 0.16 ^b^	273.35 ± 10.51 ^a^	81.29 ± 0.38 ^b^

Note: Data are presented as mean ± SD from triplicate analyses. Different superscript letters in the same column indicate significant differences between the mean values at *p* ≤ 0.05.

**Table 4:** Yield, total phenolic, and total flavonoid contents of the fingerroot extracts.

Run	*A/*Pressure (Bar)	*B/*Temperature (°C)	*C/*CO_2_ Flow Rate	*D/*Co-Solvent (% Sample)	Yield (%)	Phenolic mg GAE/g	Flavonoid (mg QE/g)
1	(1) 300	(1) 55	(−1) 1	(−1) 0	10.50	249.10	168.80
2	(−1) 200	(1) 55	(1) 3	(1) 100	31.15	262.30	254.70
3	(1) 300	(0) 45	(0) 2	(0) 50	20.25	318.80	206.80
4	(1) 300	(−1) 35	(−1) 1	(−1) 0	8.75	322.90	179.30
5	(0) 250	(0) 45	(0) 2	(0) 50	21.75	434.20	317.70
6	(0) 250	(0) 45	(0) 2	(0) 50	22.40	484.10	323.60
7	(0) 250	(0) 45	(1) 3	(0) 50	22.60	476.70	344.70
8	(1) 300	(1) 55	(1) 3	(−1) 0	8.25	274.20	114.00
9	(1) 300	(1) 55	(−1) 1	(1) 100	32.00	305.70	263.30
10	(−1) 200	(−1) 35	(−1) 1	(−1) 0	6.25	212.90	72.80
11	(−1) 200	(−1) 35	(−1) 1	(1) 100	33.78	368.90	232.70
12	(0) 250	(0) 45	(0) 2	(−1) 0	8.00	378.60	115.70
13	(1) 300	(1) 55	(1) 3	(1) 100	31.08	272.90	306.90
14	(0) 250	(0) 45	(0) 2	(0) 50	22.75	438.80	319.70
15	(0) 250	(0) 45	(0) 2	(0) 50	22.25	436.40	318.40
16	(0) 250	(0) 45	(0) 2	(1) 100	28.75	440.00	352.80
17	(0) 250	(0) 45	(−1) 1	(0) 50	21.90	429.80	226.80
18	(0) 250	(1) 55	(0) 2	(0) 50	22.05	413.60	283.40
19	(1) 300	(−1) 35	(1) 3	(1) 100	31.85	350.10	274.80
20	(−1) 200	(−1) 35	(1) 3	(−1) 0	6.75	305.50	68.30
21	(−1) 200	(0) 45	(0) 2	(0) 50	17.50	341.40	169.70
22	(−1) 200	(1) 55	(1) 3	(−1) 0	7.00	192.90	124.90
23	(−1) 200	(1) 55	(−1) 1	(−1) 0	5.75	216.90	76.20
24	(−1) 200	(−1) 35	(1) 3	(1) 100	31.65	396.40	220.80
25	(0) 250	(0) 45	(0) 2	(0) 50	23.05	434.50	322.40
26	(1) 300	(−1) 35	(1) 3	(−1) 0	12.25	220.70	157.60
27	(−1) 200	(1) 55	(−1) 1	(1) 100	29.05	271.40	242.20
28	(0) 250	(−1) 35	(0) 2	(0) 50	20.00	404.40	397.60
29	(0) 250	(0) 45	(0) 2	(0) 50	24.80	456.60	318.30
30	(1) 300	(−1) 35	(−1) 1	(1) 100	32.35	332.60	282.90

**Table 5:** ANOVA results of the response surface quadratic model for the extraction of fingerroot by supercritical fluid extraction (SFE).

Source	Sum of Squares	df	Mean Square	F-Value	*p*-Value
**Extraction Yield**					
Model	2482.91	14	177.35	57.54	<0.0001 *
Residual	46.23	15	3.08		
Lack of Fit	40.61	10	4.06	3.61	0.0846 **
Pure error	5.62	5	1.12		
Cor Total	2529.14	29			
R^2^	0.9817				
**Total Phenolic (TPC)**					
Model	2.047 × 10^5^	14	14,618.70	14.93	<0.0001 *
Residual	14,689.42	15	979.29		
Lack of Fit	12,722.29	10	1272.23	3.23	0.1036 **
Pure error	1967.13	5	393.43		
Cor Total	2.194 × 10^5^	29			
R^2^	0.9330				
**Total Flavonoid (TFC)**					
Model	2.24 × 10^8^	14	16,020.03	10.35	<0.0001 *
Residual	23,215.1	15	1547.67		
Lack of Fit	23,185.55	10	2318.56	392.33	<0.0001 *
Pure error	29.55	5	5.91		
Cor Total	2.48 × 10^8^	29			
R^2^	0.9062				

Note: * denotes a statistically significance at *p* ≤ 0.05. ** indicates a non-significt result *p* > 0.05.

**Table 6:** The relative error between the predicted and actual values for the extraction yield, TPC, and TFC of the extract under the optimized conditions.

Values	Optimized Process Parameters				Yield (%)	TPC (mg GAE/g)	TFC (mg QE/g)
	Pressure (Bar)	Temperature (°C)	CO_2_ Flow Rate (L/min)	Co-Solvent (% Sample)			
Predicted	250.103	45	3	99.99	32.51	463.600	328.905
Actual	250	45	3	100	28.67	354.578	273.479
% error	0.04	0	0	0.01	11.81	23.516	16.852

**Table 7:** Pinocembrin and pinostrobin contents in the fingerroot extracts.

Run	Pressure (Bar)	Temperature (°C)	CO_2_ Flow Rate (L/min)	Co-Solvent (% Sample)	Pinocembrin mg/g	Pinostrobin mg/g
1	(1) 300	(1) 55	(−1) 1	(−1) 0	33.97	73.82
2	(−1) 200	(1) 55	(1) 3	(1) 100	32.79	75.29
3	(1) 300	(0) 45	(0) 2	(0) 50	37.43	92.47
4	(1) 300	(−1) 35	(−1) 1	(−1) 0	20.93	65.14
5	(0) 250	(0) 45	(0) 2	(0) 50	51.06	108.62
6	(0) 250	(0) 45	(0) 2	(0) 50	51.66	107.83
7	(0) 250	(0) 45	(1) 3	(0) 50	58.97	112.59
8	(1) 300	(1) 55	(1) 3	(−1) 0	40.58	85.36
9	(1) 300	(1) 55	(−1) 1	(1) 100	38.08	82.14
10	(−1) 200	(−1) 35	(−1) 1	(−1) 0	14.56	87.25
11	(−1) 200	(−1) 35	(−1) 1	(1) 100	37.44	94.18
12	(0) 250	(0) 45	(0) 2	(−1) 0	42.41	88.47
13	(1) 300	(1) 55	(1) 3	(1) 100	30.93	76.91
14	(0) 250	(0) 45	(0) 2	(0) 50	50.37	108.19
15	(0) 250	(0) 45	(0) 2	(0) 50	48.87	107.95
16	(0) 250	(0) 45	(0) 2	(1) 100	62.10	115.28
17	(0) 250	(0) 45	(−1) 1	(0) 50	44.71	95.63
18	(0) 250	(1) 55	(0) 2	(0) 50	41.51	89.42
19	(1) 300	(−1) 35	(1) 3	(1) 100	13.93	82.75
20	(−1) 200	(−1) 35	(1) 3	(−1) 0	17.47	92.31
21	(−1) 200	(0) 45	(0) 2	(0) 50	24.66	96.84
22	(−1) 200	(1) 55	(1) 3	(−1) 0	61.91	88.12
23	(−1) 200	(1) 55	(−1) 1	(−1) 0	36.45	91.27
24	(−1) 200	(−1) 35	(1) 3	(1) 100	8.27	84.69
25	(0) 250	(0) 45	(0) 2	(0) 50	56.96	109.52
26	(1) 300	(−1) 35	(1) 3	(−1) 0	10.93	79.46
27	(−1) 200	(1) 55	(−1) 1	(1) 100	9.59	99.37
28	(0) 250	(−1) 35	(0) 2	(0) 50	42.93	89.58
29	(0) 250	(0) 45	(0) 2	(0) 50	58.33	108.71
30	(1) 300	(−1) 35	(−1) 1	(1) 100	61.91	93.24

## Data Availability

The original contributions presented in the study are included in the article, further inquiries can be directed to the corresponding author.
